# Incorporating fantasy into gamification promotes student learning and quality of online interaction

**DOI:** 10.1186/s41239-022-00335-9

**Published:** 2022-06-14

**Authors:** Shurui Bai, Khe Foon Hew, Donn Emmanuel Gonda, Biyun Huang, Xinyi Liang

**Affiliations:** 1 Faculty of Education, The University of Hong Kong, Pok Fu Lam, Hong Kong SAR; 2 Centre for the Enhancement of Teaching and Learning, The University of Hong Kong, Pok Fu Lam, Hong Kong SAR; 3 Centre for Learning Sciences and Technologies, The Chinese University of Hong Kong, Shatin, Hong Kong SAR; 4 Technology-Enriched Learning Initiative, The University of Hong Kong, Pok Fu Lam, Hong Kong SAR; 5 The University of Hong Kong, Room 322, Runme Shaw Building, Pokfulam Road, Pok Fu Lam, Hong Kong SAR

**Keywords:** Gamification, Fantasy, Narrative, Fully online learning

## Abstract

We used the design-based research approach to test and refine a theoretically grounded goal-access-feedback-challenge-collaboration gamification model. The testbed was a 10-week, university-level e-learning design course offered in two consecutive semesters. In Study 1, we implemented the initial goal-access-feedback-challenge-collaboration model in semester one of the 2020–2021 academic year (*N* = 26). The aim was to enhance student behavioral engagement in online discussion forums, affective engagement in the class, and learning performance. The results of Study 1 showed that although most participants were engaged in this gamified learning experience during the first two sessions, they gradually lost interest and their participation in online discussions dropped over the next eight weeks. Thus, we introduced a new element, *fantasy*, into the original model. In Study 2, we tested the effectiveness of the goal-access-feedback-challenge-collaboration-fantasy model on students’ learning outcomes in semester two of 2020–2021 (*N* = 23). The results of Study 2 suggested that, compared to the original model, the goal-access-feedback-challenge-collaboration-fantasy model can better promote students’ engagement in online discussion, as measured by increased interaction with peers, learning experience, and learning performance.

## Introduction

The COVID-19 pandemic has forced many higher education institutions to fully embrace online teaching. Regrettably, fully online teaching is often regarded as a less potent option (Hodges et al., 2020) and as less satisfying (Wang et al., 2019) than in-person teaching. In a survey of more than 400 college students who have attended online classes, the participants reported that the lack of in-person interaction and the lack of motivation to get started on coursework are the two main challenges of their online classes (Friedman, 2020). Andrew et al. (2021) suggested that when teachers cannot observe them directly, students can easily become disengaged in fully online classes. In this study, even though students were present during the online class, they were not participating in the class discussion but were merely “lurking” (Andrew et al., 2021).It is even more challenging to engage students in a fully online class over a long period (Saqr & López-Pernas, 2021). Therefore, the challenge of engaging learners continuously in a fully online class remains very relevant.

Gamification has often been applied in education to promote learners’ motivation in completing lesson activities. Yet, despite the growing interest in gamified classes, evidence of its ability to enhance student motivation is not yet consistent (e.g., Koivisto & Hamari, 2019; Zainuddin et al., 2020). For instance, some studies have shown that gamification, in the form of awarding learners with badges and experience points, can help promote student participation in course activities (Zahedi et al., 2021) or active participation in answering questions on the screen (Baydas & Cicek, 2019). However, other studies have reported a negative or inconclusive effect on student learning outcomes (Koivisto & Hamari, 2019). Therefore, gamification is not effective per se (Sailer et al., 2017). Instead, the design of effective gamified practice needs to be grounded in the theoretical understanding of the motivational mechanisms through which gamification attains its impact (Krath et al., 2021). However, gamification research lacks theoretical foundations, which leads to a partial view of gamification as well as shortcomings in the research designs of relevant investigation (Koivisto & Hamari, 2019). Therefore, it is important for researchers to investigate the underlying theories and how they relate to motivation so that gamification practice can be designed to achieve the desired results (Krath et al., 2021).

In this study, we used the goal-access-feedback-challenge-collaboration (GAFCC) gamification model first developed by Huang and Hew (2018) to guide our initial gamification design. We chose this model because of its reported effectiveness in enhancing student engagement and learning performance in university on-campus courses (Huang & Hew, 2018; Huang et al., 2019) as well as its comprehensive theoretical foundations based on five motivational theories (see Sect. 2.1 for details).

We conducted design-based research (DBR) in two consecutive cycles of the same fully online postgraduate course. First, we implemented the GAFCC model in semester one of the 2020–2021 academic year (Study 1, *N* = 26), seeking to enhance student behavioral engagement in online discussions, affective engagement in the class, and learning performance. The results of Study 1 showed that although most participants were engaged in this gamified learning experience during the first two sessions, they gradually lost interest after becoming familiar with the gamification scheme. Student participation in online discussions dropped over the next eight weeks.

Thus, drawing on students’ responses and the literature, in the second study (Study 2), we introduced a new element, *fantasy*, into the original model (GAFCC-F). “Fantasy” is a motivational factor proposed by Malone (1981) to afford players’ fulfillment of wishes by providing a virtual world that is detached from reality. In an educational setting, fantasy has often been used in the form of narratives (Aldemir et al., 2018) or narrative-based scenarios to engage learners (Jagušt et al., 2018) and immerse them in virtual activities. We tested the effectiveness of the GAFCC-F model on students’ learning outcomes in semester two of 2020–2021 (*N* = 23). The results of Study 2 suggested that, compared to the original GAFCC model, the GAFCC-F model can better promote students’ engagement in the online discussion, as measured by increased interaction with peers, learning experience, and learning performance.

## Related literature

### GAFCC gamification design model: theory and application in practice

The GAFCC model is grounded in five motivation theories: goal-setting theory (Locke & Latham, 2002), flow theory (Csikszentmihalyi, 1990), self-determination theory (Deci & Ryan, 2000), social comparison theory (Festinger, 1954), and behavioral reinforcement theory (Skinner, 1965). We briefly describe the five motivational elements (i.e., goal, access, feedback, challenge, and collaboration) of our study below.

The first element involves helping students set up long- and short-term goals by establishing rewarding game elements, such as participation-based badges and experience points. The second element, access, involves offering students the autonomy to choose challenges with various levels of difficulty. The third involves providing instant feedback on students’ performance in learning activities to engage them in self-evaluation. The fourth involves challenging students by giving them opportunities to compete with themselves or their peers. Finally, collaborative tasks allow learners to share goals and interact with each other more often.

Huang and Hew (2018) conducted two quasi-experiments that compared the effects of gamified flipped courses using the GAFCC design model with those of non-gamified flipped courses on student completion rates and on the quality of pre-class and post-class activities and perceptions. In both experiments, the students in the gamified flipped class completed more activities of higher quality than students in the control class. Furthermore, the students gave overall positive comments on the gamified learning experience. Another empirical study using the GAFCC model revealed that students in a gamified flipped class performed better than students in a non-gamified flipped class, as measured by pre-class thinking activities and a learning performance test (Huang et al., 2019). Huang et al. (2019) also examined how a GAFCC-supported gamified course affected students’ peer-feedback quality and interactions in online discussion forums. The results suggested that the gamified group had a denser social network than the control group; learners in the gamified group showed higher critical thinking skill levels than those in the control group. In summary, preliminary research suggested that the GAFCC model is effective in promoting students’ learning performance, engagement in out-of-class activities, and peer feedback in discussion forums and that students perceive gamified learning supported by this design model positively. Nevertheless, despite these positive findings, this model has not yet been applied in a fully online teaching context. Fully online courses are generally associated with higher levels of student disengagement than on-campus courses (Xavier & Meneses, 2020). Whether the original GAFCC model can motivate fully online students remains an open question.

### Fantasy as a motivational factor

Fantasy was first proposed by Malone (1981) as a motivational factor to afford players’ fulfilment of wishes by providing a virtual world that is detached from reality. Malone and Lepper (1987, p. 240) define fantasy as an environment that “evokes mental images of physical or social situations not actually present.” Disneyland is a typical example of a representation of fantasy that is emotionally appealing to visitors. The embedded narrative in a given environment is an important element in the creation of fantasy sensations (Shi & Shih, 2015). The narrative-based fantasy is usually pre-generated, existing before the player has any interaction with it. The aim of a narrative-based fantasy is to provide the player with a sense of purpose for each action in the game (Salen & Zimmerman, 2003).

Malone and Lepper (2021) suggested that embedding educational activities into a fantasy context enhances students’ learning motivation and engagement. Previous empirical studies revealed that narrative-based fantasy can be a powerful force in facilitating students’ learning motivation, learning performance (Cordova & Lepper, 1996; Parker & Lepper, 1992), and affective engagement (Islas Sedano et al., 2013). Learners who are immersed in an imaginary world sustained by a video game are easily emotionally engaged in the learning environment and its content (Islas Sedano et al., 2013).

Two gamification interventions have used narrative-based fantasy to enhance student learning outcomes. First, Jagušt et al. (2018) proposed a virus-fighting setting, presenting the following narrative to students prior to taking quizzes in the face-to-face class: “In the previous lesson, we fought and won a battle against small viruses on our tablets, but there is still the big mother-virus in our computer servers….” (Jagušt et al., 2018, p. 499). The results indicated that using a narrative-based fantasy with leaderboards and a points system leads to enhanced learning outcomes. The authors also suggested that gamification can be done offline by simply adding narrative-based fantasy as an opening to an activity. Second, Aldemir et al. (2018) carried out a qualitative study to explore students’ overall perceptions of various game elements in a “wizarding world”. The authors created a narrative-based fantasy based on the Harry Potter series, setting up a magical school with four houses. The participants could start their course as apprentices and become masters by the end of the semester. In-class sessions were also offered to guide students’ progress in the story. The participants’ interview responses confirmed the potential positive effects of narrative-based fantasy on student learning motivation and that immersion in the “wizarding world” was a joyful learning experience.

However, how narrative-based fantasy affects student course engagement in a fully online learning setting where there are no teachers to guide the students in completing learning tasks remains unknown. The research questions that guided this study were as follows:

Research question 1: What are students’ and teachers’ reflections on using a GAFCC gamification model? Does the GAFCC gamification model require any refinements?
Research question 2: How well does the GAFCC-F model enhance students’ learning performance and online social interaction compared to the original GAFCC model?
Research question 3: What are students’ and teachers’ perceptions of the GAFCC-F model?

## Method

We adopted the DBR approach, which is designed for researchers and educators who intend to increase the effects of educational research and improve real-world classroom practices (Anderson & Shattuck, 2012). There are three main phases of DBR: (a) preparing the experiment and placing it in a theoretical context, (b) applying the interpretive framework and conducting the experiment to support learning, and (c) performing retrospective analyses (Reimann, 2011). DBR enables practitioners to focus on designing, testing, and refining a theory-based intervention by addressing practical educational problems in several iterations (Collins et al., 2004; Jan et al., 2010).

We carried out the three phases of DBR over two studies. First, we implemented the initial GAFCC gamification design model in semester one of the 2020–2021 academic year (Study 1, *N* = 26). Second, we refined the GAFCC model based on the challenges revealed in the data, addressing them by introducing a new element, *fantasy*. Our design used fantasy to amplify the effects of the original five elements (i.e., goal, access, feedback, challenge, and collaboration) and address decreased affective and behavioral engagement in students. Third, in semester two of 2020–2021, we tested the effectiveness of the new GAFCC-F gamification design model on students’ learning performance, interaction in online discussion forums, and perceptions by comparing these outcomes to those of the GAFCC design model (Study 2, *N* = 23) (see Sect. 3.2 for the design rationale of the GAFCC-F model). Studies 1 and 2 were conducted in the same online course, “E-learning Strategies and Management” (course length: one semester, or 10 weeks) offered in two successive semesters. These two studies were carried out during the COVID-19 pandemic; therefore, all the courses were delivered via a fully online synchronous teaching mode via Zoom, a web-based videoconferencing platform.

We aimed to compare the effects of the GAFCC and GAFCC-F gamification design models on students’ learning performance, interaction in online discussion forums, and perceptions. The two courses were taught by the same instructors using the same courseware, assessment rubrics, and final assignments. All the learning materials and tasks were distributed by the same learning management system, Moodle. The participants in the two studies shared an interest in technology-enhanced learning, as they were admitted to the same program. “E-learning Strategies and Management” is a postgraduate-level elective course with no prerequisites for students’ enrolment. The course introduced six specific learning outcomes (i.e., factual learning, conceptual learning, problem-solving, procedural learning, principle learning, and attitude learning) as well as the relevant instructional strategies to facilitate mastery of the six learning outcomes in the e-learning context.

Ethics approval for this study was obtained from the Institutional Review Board of the authors’ university. The participants were informed of the research objectives, and they all signed the consent forms before the intervention.

### Study 1: Implementation of the GAFCC gamification design model

#### Participants

Twenty-six postgraduate students (18 females, eight males) participated in Study 1. Their ages ranged from 22 to 48 years (*M* = 28.5, *SD* = 6.19). Among them, 92.3% (24 out of 26) were from East Asia (i.e., Hong Kong and Mainland China) and two participants were from the United Kingdom.

#### Settings of the GAFCC gamified class

**Fig. 1:**
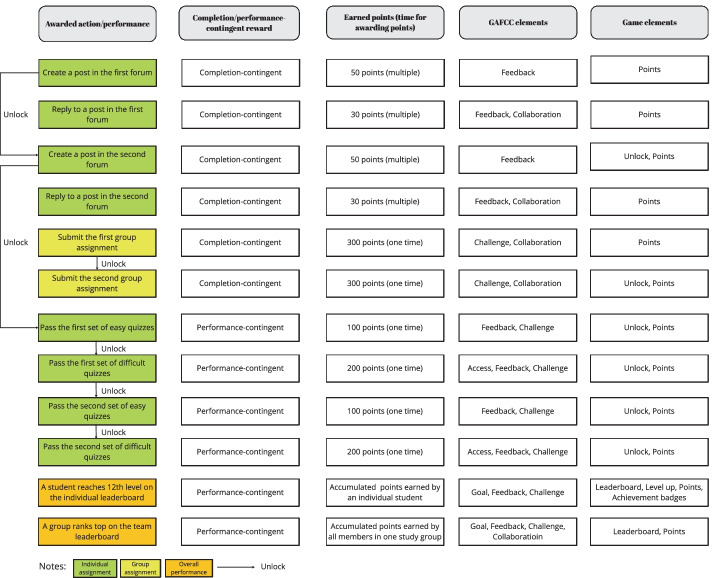
Points-adding system used in the GAFCC group (Study 1)

Study 1 was designed using the GAFCC gamification design model. We managed all the learning materials and activities on Moodle. A plugin, “Level Up” (Sinnott & Xia, 2020) was installed in Moodle to set up the gamified environment. “Level Up” logged students’ real-time activity results according to pre-specified rules in the system. According to these rules, students were automatically granted a certain number of points when they accomplished specific actions and reached certain performance measures. An individual leaderboard displayed individual students’ accumulated points and their respective ranking determined by those points, whereas a team leaderboard displayed the accumulated points earned by all members in one study group and the study group’s ranking. We announced the points-adding rules at the first session of the course (see Fig. 1).

The implementation of the course using the GAFCC model was conducted as follows. First, in the first session of the course, we assigned a *goal* for the students, which was to reach the 12th level by completing the various learning tasks in Moodle by the end of the semester. Second, we used the “unlock function” to activate the *access* element, meaning that the learning tasks on Moodle were not designed to be completed at one go. Instead, students were required to complete the available individual easy quizzes, create a post in the first discussion forum, and submit the first group assignment before moving on to subsequent tasks (i.e., individual difficult quizzes, second discussion forum, and second group assignment).

Third, we gave students’ instant *feedback* on their performance on quizzes, posts on discussion forums, and submissions of group projects by automating the points-adding process for each task (see Fig. 1 for points values). Points were given to recognize students’ efforts in completing the learning tasks. Two types of incentive schemes were used. The first was a performance-contingent point system, wherein points would only be given once a student had reached a passing grade on a given quiz. The second was a completion-contingent point system, in which points would be given when a student had completed a task (e.g., posts, group assignments).

Fourth, to introduce *collaboration*, we assigned four group assignments during the semester. Each group assignment required the members to propose relevant instructional strategies to teach a certain topic. For example, students were asked to work in groups and propose appropriate instructional strategies to teach the concept of fruits. Fifth, we assigned several sets of difficult quizzes and difficult group assignments, so students could *challenge* themselves by attempting these tasks after they had accomplished the easier ones.

### Feedback on the GAFCC gamified learning experience

We collected the 24 students’ reflections on gamified learning at the end of the semester. Most students (97%) preferred gamified learning to their previous, conventional, lecture-based courses. However, many students (67%) stated that although they were very engaged in this gamified learning experience in the first two sessions, they gradually lost interest when they became familiar with the gamification scheme.

**Fig. 2:**
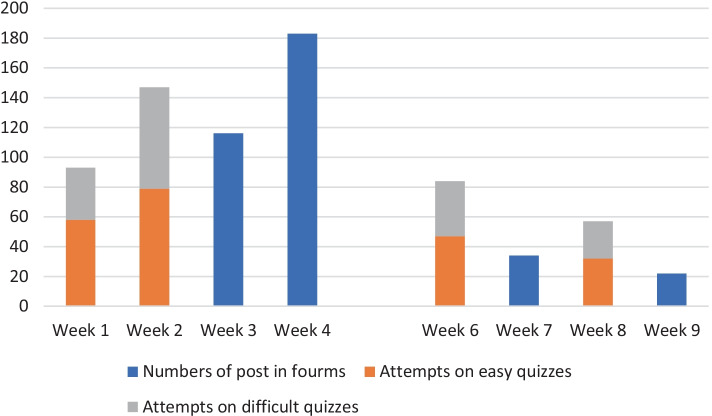
Number of students’ posts in forums and attempts on easy and difficult quizzes in the GAFCC model

Additionally, we examined changes in students’ participation, such as posting on forums and attempting quizzes, over the 10 weeks. The results showed that the number of posts and attempts decreased sharply after Week 5 (halfway through the intervention). The number of posts in the first two forums, released before Week 5, were 116 and 183; however, the number of posts in the last two forums, released after Week 5, were 34 and 22. The average number of attempts on the first two sets of easy and difficult quizzes was 68.5 and 51.5, respectively. The average number of attempts on the last two sets of easy and difficult quizzes was 39.5 and 31, respectively (see Fig. 2). Some of the students (45%) who were frequent video game players stated that this gamification would have provided a more exciting learning experience if it included a virtual play environment, for example, virtual characters and a back narrative for each gate/level, thus simulating the thrilling experience of playing a video game.

In addition, instructors noted that the students became less interested in completing new tasks after the novelty period of the first three weeks. One instructor suggested setting up a narrative to link all the tasks, permitting students to follow this narrative to unlock tasks and thus making passing each level more meaningful. It was proposed that the students may obtain a sense of achievement in completing all the tasks and accomplishing the final mission at the end of the semester.

### Study 2: Implementation of the GAFCC-F gamification design model

The main problems we encountered in using the GAFCC model were students’ decreased affective engagement (i.e., students became less interested in this gamified learning) and behavioral engagement (i.e., students displayed less interaction with peers on discussion forums and fewer attempts on quizzes) over time. In response, we introduced a new element, *fantasy*, originating from the literature on computer games, into the GAFCC model. We created a fantasy world by combining certain elements from reality (e.g., tourist attractions in City K, COVID-19 background) and certain imaginative elements (e.g., a talking dragon in the “Save Princess Joanne” story, see details in Sect. 3.4.2).

#### Participants

**Fig. 3:**
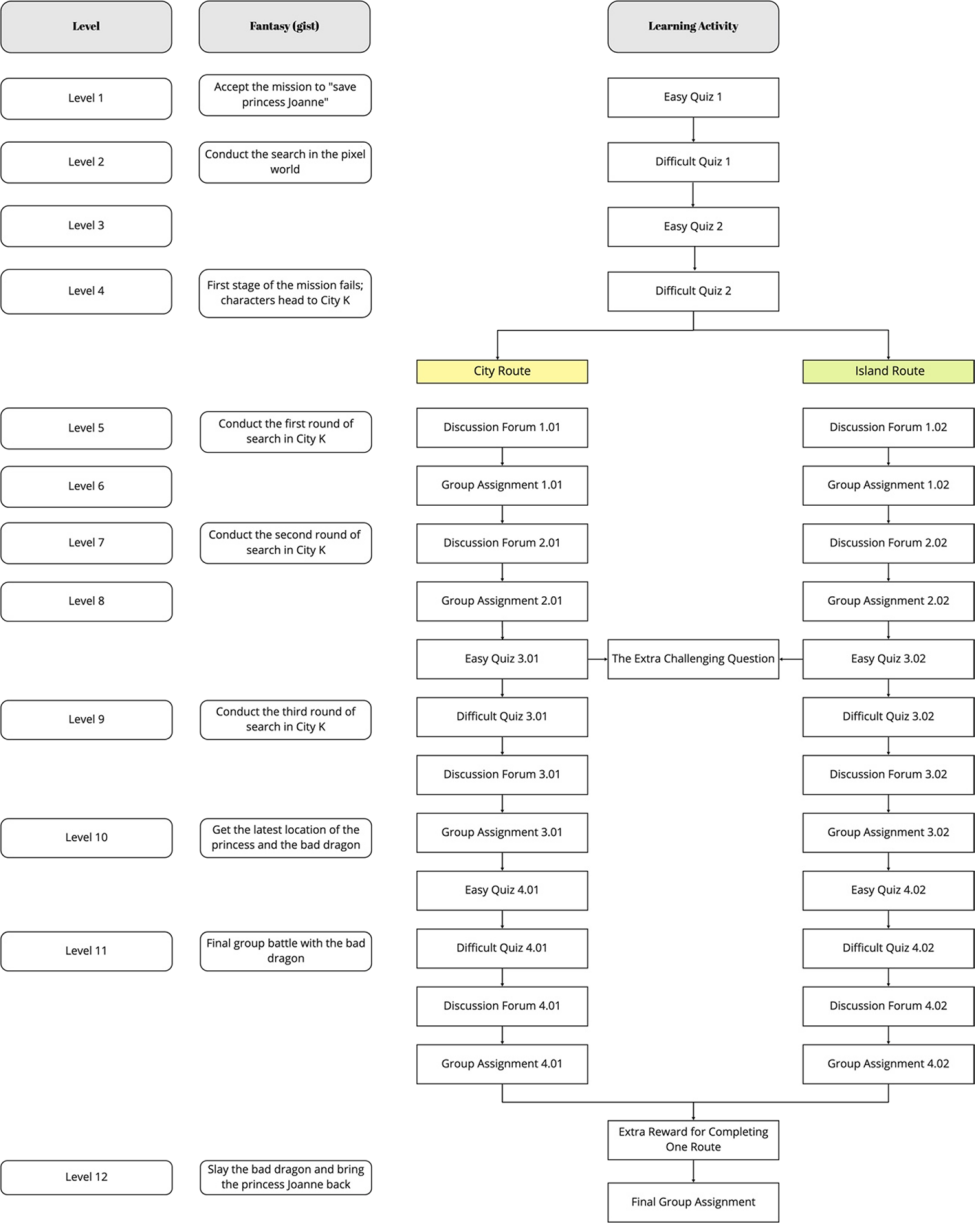
Map of fantasy gamified learning activities levels

Study 2 involved 23 postgraduate students (15 females and 8 males). The ages of the participants ranged from 22 to 47 years (*M* = 26.35, *SD* = 5.93) (Fig. 3).

#### Settings of the GAFCC-F gamified class

**Fig. 4:**
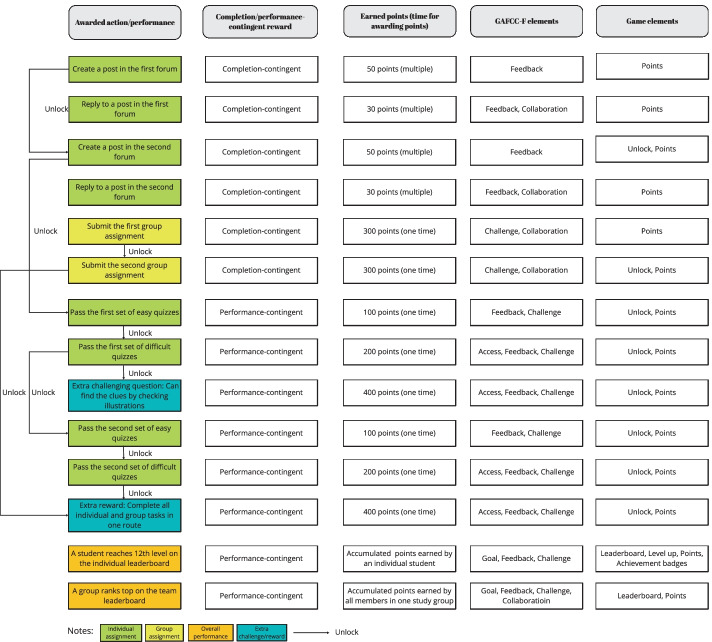
Points-adding rules used in the GAFCC-F group (Study 2)

**Fig. 5:**
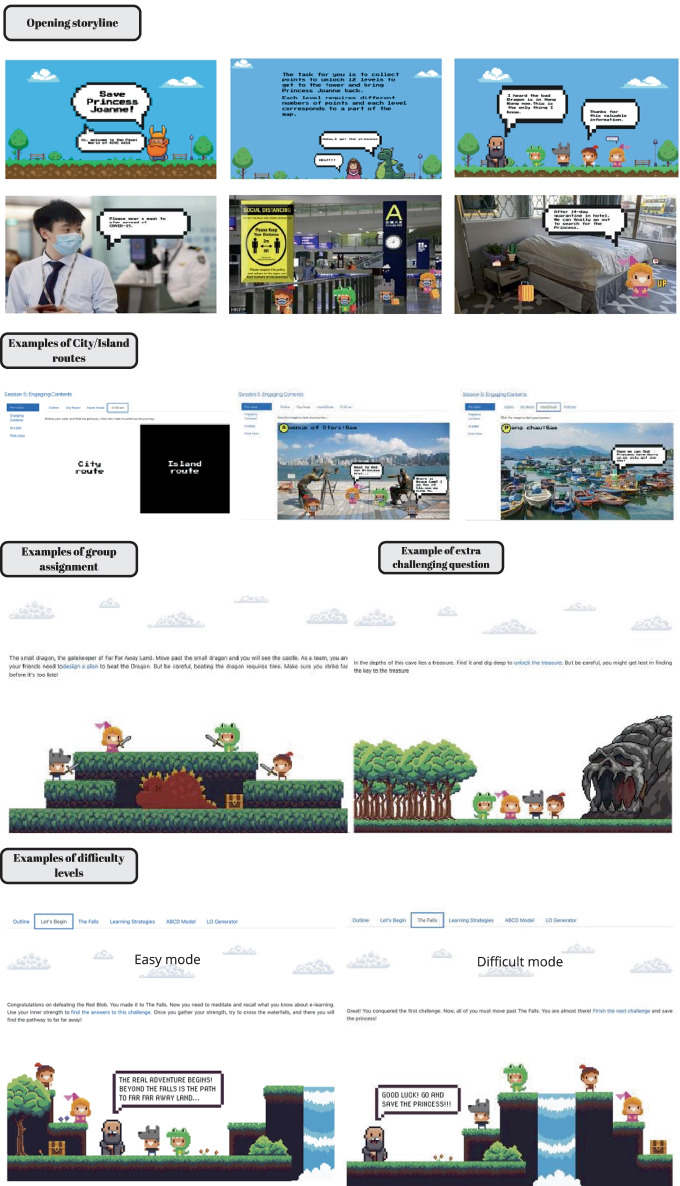
Fantasy and embedded narrative in individual quizzes, group assignments, and challenging questions

This section describes how we implemented the fantasy element in the GAFCC-F model. Figure 4 lists the points-adding rules and game elements of this model. We introduced the game rules and the backstory of “Save Princess Joanne” in the first session of the course. The goal of this story (i.e., to rescue Princess Joanne from the bad dragon) was the core concept of this round of gamification design. We also set up a lead-in backstory to explain why the game characters (i.e., the course students) were required to travel to City K (an authentic city) to save the princess. We created an elderly character, who was embodied in the instructors, to announce the points-adding rules and main tasks in the narrative. Figure 5 presents images of the “Save Princess Joanne” narrative-based fantasy in the imaginary pixel world.

The students’ final mission was to collect points and unlock 12 levels to bring Princess Joanne safely back at the end of the semester. Each level required a certain number of points, and each level corresponded to a part of the game map (see Fig. 3). First, students were required to choose a character out of four pre-set characters to activate the adventure. Second, to meet students’ need for autonomy, we offered two routes (i.e., the City or Island Route) to choose from. The plot of each level could only be unlocked when students had satisfied the previous task. Among the 12 levels, we assigned two very challenging questions for those who were interested in gaining a large number of points in one go. We also set up a rewards section, where students could collect 400 points by completing all the tasks of one route. The remaining rules of play were identical to those of Study 1 (see Fig. 4 for details).

### Measures in study 1 and 2

We measured the students’ learning performance, interaction with peers in online discussion forums, and perceptions by using the same instruments in both studies.

#### Students’ learning performance

To measure the students’ learning performance, a pre-intervention test, a mid-term test, and a post-intervention test were administered. The pre-intervention test was a voluntary performance test, students could choose not to take it. The pre-intervention test contained seven short essay questions examining students’ prior ability to name an appropriate instructional strategy to obtain a given learning outcome. This test was conducted in the first session of the course. The following are sample questions from the test: *What are some important elements of a good lesson objective? What are the instructional strategies to teach “concepts”? What are the instructional strategies to teach “facts”?*

We measured the students’ learning performance with a mid-term exam (i.e., at Week 5). The mid-term test was also a voluntary performance test. This test examined both the students’ factual knowledge (i.e., explanations of concepts and differentiation of several e-learning design models) and problem-solving ability (i.e., application of appropriate instructional strategies to address a real-world instructional scenario).

The following are sample questions testing factual knowledge: *What are the differences between learning outcomes and learning objectives? What are the differences between the ADDIE and 5E models?* A problem-solving, scenario-based activity required students to design a fully online course. First, students were required to identify specific learning outcomes and write corresponding learning objectives for a given scenario. Second, they were required to list key instructional strategies to achieve the learning objectives. Finally, they were asked to list all the content and technologies needed to complete the course design under the guidance of the 5E model (i.e., engage, explore, explain, elaborate, and evaluate). The scenario description was as follows: *You are working in an instructional design company, and you have been assigned to design a course. The client asked you to design a fully online, five-day, asynchronous course to train 20 newly recruited, inexperienced insurance agents. The course will help the new employees master information about three insurance packages and two sale techniques.*

The post-intervention test referred to the following three final assignments: a) design a storyboard to train customer-facing representatives to sell one beauty product, b) analyze one e-learning hack, and c) design and build content for one online Moodle course.

#### Students’ interaction with peers in online discussion forums

We downloaded the logs of students’ posts in the four online discussion forums generated in the two studies. The first online discussion forum (DF 1) was about the students’ writing their own learning objectives. The students were asked to come up with two scenarios in which their written learning objectives could be applied. The second online discussion forum (DF 2) invited students to share their opinions on how the Fourth Industrial Revolution could influence the education sector. They were asked to select at least two of eight essential technologies listed on the designated website and discuss how they could integrate these technologies into teaching and learning. The third discussion forum (DF 3) was about how the students, as instruction designers, would address some of the issues brought about by COVID-19. The students were required to share a link to an article about how schools, universities, or companies are tackling the issue of teaching and learning or corporate training under these circumstances. From the article they posted, they were asked to identify the key strategies being implemented in these institutions. The fourth discussion forum (DF 4) invited students to share one e-learning application and suggest a scenario in which it could be used. The students’ performance on discussion forums did not contribute to their final course grades, as participation on the forums was not compulsory.

We examined the quality of students’ online posts (both their own posts and their comments on peers’ posts), using the two rating scales for assessing collaborative online notes proposed by van Aalst (2009), *knowledge quality* and *significance of findings*. Knowledge quality is an assessment of the epistemic position of the knowledge, scored as simple conjecture (with a score of 1), a factual claim (2), a partly integrated explanation invoking at least one concept (3), or a comprehensive explanation invoking multiple concepts (4). Significance of finding is an assessment of how well students identify the knowledge that they have learnt. Comments were scored as a restatement of the knowledge (with a score of 1), a clear description of the knowledge without limitations (2), a profound description of the knowledge with some limitations (3), or a comprehensive description of the knowledge with limitations and inquiry to others (4) (see van Aalst, 2009 for details).

We further used directed content analysis to code students’ post content, assessing how they interacted with their peers in a fully online social community. Directed content analysis provides a structured process in coding (Hickey & Kipping, 1996), permitting us to use pre-determined codes from prior research to start the coding immediately (Hsieh & Shannon, 2005). A new code was generated if the text did not fit in the initial coding scheme (Hsieh & Shannon, 2005). We adopted van Aalst’s (2009) sub-themes codes for the “community” aspect as the guide for our content analysis, as our study had a similar data source (i.e., students’ posts on online discussion forums) and the same main theme of “community.” There were seven initial sub-codes, namely *apologizing*, *co-authoring*, *innovating*, *giving credit*, *deciding*, *encouraging*, and *seeking views*. We created new codes or removed codes as needed to fit the content of the texts. Two raters independently marked the two rating scales and coded the content of posts. All the discrepancies were discussed until full agreement was reached.

To understand students’ online interactive patterns, a social network analysis approach was applied to analyze the students’ posts in all four online discussion forums. We used UCINET as the analytical tool (Borgatti et al., 2002). The five indicators we examined were *network size*, *density*, *degree centralization*, *out-degree*, and *in-degree centrality*. “Network size” refers to the number of actors in the network (Collins & Clark, 2003). “Network density” refers to the prevalence of direct ties in a network graph (Frey, 2018). This indicator helps to analyze the connectedness within a network (Scott, 2012). Centralization indicates how centered a network is around the most active nodes (Claros et al., 2016). A high value of centralization suggests that interaction is concentrated on a few actors, in other words that not many people contribute to an online discussion (Claros et al., 2016). In contrast, a low value of centralization indicates that many students contributed equally to the online discussion (Huang et al., 2019). Out-degree and in-degree centrality were used to identify how active the students were in posting and commenting. The out-degree value indicates the sum of connections from a given node toward other nodes, and the in-degree value indicates the sum of connections from other nodes directed toward a node (Hansen et al., 2011). For example, in an online discussion forum, “out-degree” refers to the number of posts that a student sent out in the community and “in-degree” refers to the number of replies that a student received in the community.

#### Students’ and teachers’ perceptions

To understand the students’ and teachers’ overall perceptions of the GAFCC-F model, we conducted two rounds of semi-interviews at the end of the semester, interviewing 15 students and two teachers. Sample questions are as follows: *What do you think of “Save Princess Joanne in City K” in terms of motivating or demotivating you in completing individual work? What do you think of “Save Princess Joanne in City K” in terms of motivating or demotivating you in collaborating with group members?* and *do you have any suggestion for improving this learning experience?* When interviewing the teaching team, we asked their findings based on class observation, their perceptions of the fantasy element, and their suggestions for future course design.

We used a thematic analysis approach to process the students’ responses. Several relevant themes developed via initialization, construction, rectification, and finalization (Vaismoradi et al., 2016). Two authors first independently coded the interview transcripts, then discussed the results until a mutual agreement was reached.

## Results

### Students’ learning performance results

Twenty-two students took the pre-intervention test, and all 23 students took the mid-term and post-intervention tests. We first examined whether the students in the two groups differed significantly in their prior knowledge level. After the normality of the pre-test scores data and the assumption of homogeneity of variances were verified, an independent samples t-test was conducted and no significant difference was detected (*t* (46) = -0.54, *p* = 0.595). In summary, there was no significant difference between the two design model groups in terms of the students’ initial knowledge. The descriptive statistics revealed that the students in the GAFCC-F model group had a higher mean mid-term score (M = 69.13, SD = 14.29) and post-test learning performance score (M = 83.52, SD = 7.35) than the students in the GAFCC model group (see Table 1 for details). After satisfying the normality of the sampled data and the assumption of homogeneity of variances, we further checked the mid-term and post-test scores data and found significant differences between the two groups (*t* (47) = -5.02, *p* < 0.001 [mid-term]; *t* (47) = -2.26, *p* = 0.028 [post-test]). In sum, the participants in the GAFCC-F group showed significantly higher learning performance levels than those of the GAFCC group.

**Table 1:** Students’ learning performance in two gamification design model groups (learning performance: 100 full mark)

Stage	Group	N	Mean	SD
Pre-test	GAFCC-F model	22^a^	24.27	11.11
	GAFCC model	26	22.58	10.77
Mid-term	GAFCC-F model	23	69.13**	14.29
	GAFCC model	26	42.50	21.56
Post-test	GAFCC-F model	23	83.52*	7.35
	GAFCC model	26	77.82	9.89

^a^One student chose not to take the pre-intervention test. The total number of students in GAFCC-F group was 23

**p < 0.001, *p < 0.05

### Students’ interaction with peers in online discussion forums

#### Quality and coding of students’ online posts

A small difference was observed between the two groups in the quantity of online posts (80 for the GAFCC-F group and 73 for the GAFCC group). Table 2 lists the descriptive statistics of the scores of the two scales and the quantity of the two groups’ posts. Regarding the knowledge quality, four independent-samples t-tests were conducted. The GAFCC-F group outperformed the GAFCC group significantly in DF 1 (*M* = 1.97, *SD* = 0.94), DF 2 (*M* = 3.35, *SD* = 1.04), and DF 3 (*M* = 2.66, *SD* = 0.97). As for the significance of the findings, the mean score in the GAFCC-F group was significantly higher than that of the GAFCC group for DF 2 (*M* = 3.18, *SD* = 0.99), and DF 3 (*M* = 2.74, *SD* = 0.92). Two raters independently marked the two scales, and the inter-rater reliability was 85%.

**Table 2:** Total number, knowledge quality, and significance of findings of posts in the two gamification design models

Variable	Discussion forum	Group	N	Mean	SD
Total posts without duplications	All four DFs	GAFCC-F model	80	4.13	2.45
		GAFCC model	73	4.86	2.98
Knowledge quality	DF 1	GAFCC-F model	92	1.97**	0.94
		GAFCC model	133	1.67	0.70
	DF 2	GAFCC-F model	51	3.35**	1.04
		GAFCC model	34	1.88	0.81
	DF 3	GAFCC-F model	35	2.66**	0.97
		GAFCC model	22	1.55	0.96
	DF 4	GAFCC-F model	116	2.22	1.09
		GAFCC model	142	2.16	1.04
Significance of findings	DF 1	GAFCC-F model	92	1.99	1.12
		GAFCC model	133	1.70	0.81
	DF 2	GAFCC-F model	51	3.18**	0.99
		GAFCC model	34	2.29	1.03
	DF 3	GAFCC-F model	35	2.74*	0.92
		GAFCC model	22	2.09	0.97
	DF 4	GAFCC-F model	116	2.34	1.20
		GAFCC model	142	2.29	1.04

*DF* discussion forum

**p < 0.001, *p < 0.05

Eight sub-theme codes were identified in the content analysis: *summarizing*, *apologizing*, *giving credit and appreciation*, *encouraging and agreeing*, *asking for clarification*, *seeking views*, *elaborating and giving examples*, and *reflecting and evaluating.* The GAFCC-F group had more posts elaborating and giving examples (N = 46) and reflecting and evaluating (N = 68) than the GAFCC group (see Table 3). According to the rubric created by (Christopher et al., 2004) for the evaluation of online discussion responses, adapted from Bloom’s taxonomy (Bloom, 1956), elaborating entails medium levels of thinking, whereas reflecting and evaluating entails high levels of thinking. A medium level of thinking requires students to apply what they have learned in a new way (Christopher et al., 2004). A high level of thinking requires students to combine their knowledge in a new domain and assess the value of a given idea (Christopher et al., 2004). In contrast, the GAFCC-F group had fewer summarizing posts than the GAFCC group. Summarizing involves a low level of thinking, with students restating old information (Christopher et al., 2004). The fifth author coded the posts independently and the inter-rater reliability of the two authors was 89%.

**Table 3:** Coding of posts in the two gamification design models (original posts and peer-feedback)

Sub-theme	GAFCC-F model	GAFCC model
Summarizing	37	102
Apologizing	2	4
Giving credit & appreciation	54	46
Encouraging & agreement	20	44
Asking clarification	25	32
Seeking views	12	12
Elaborating & giving examples	46	35
Reflecting & evaluating	68	42

#### Students’ online interactive pattern

**Fig. 6:**
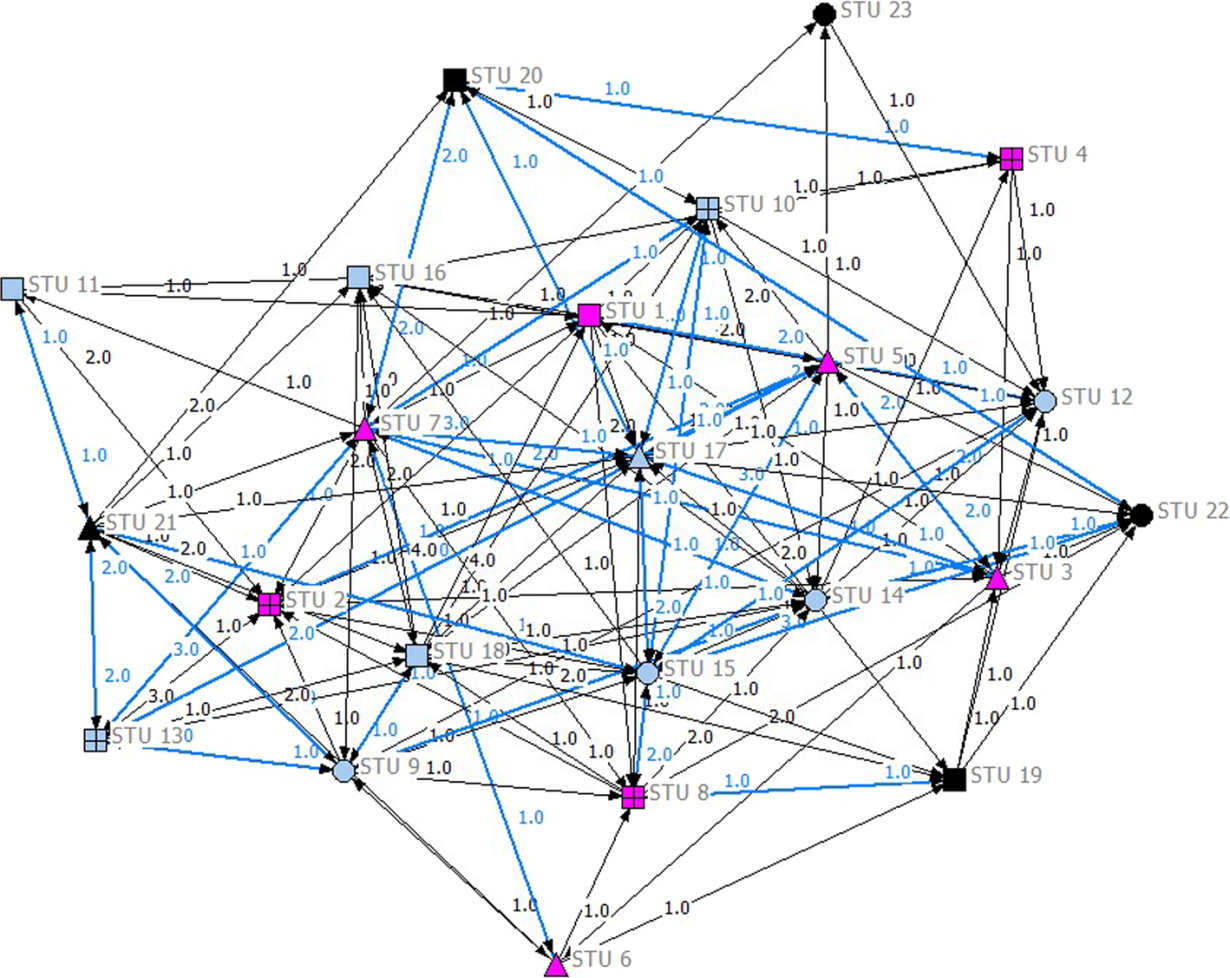
Social network interaction pattern for the GAFCC-F group (including original posts and peer comments). *Notes The pink nodes indicate students with final scores from 85 to 100 (A grade); the blue nodes indicate students with final scores from 75 to 84 score (B grade); the black nodes indicate students with final scores from 60 to 74 (C grade). Blue ties refer to reciprocal ties, and black ties are non-reciprocal ties. There were four study groups in this course, all formed by students exercising free choice. A circle (open circle) indicates Group 1; a square (open square) indicates Group 2; an upwards pointing triangle (open triangle) indicates Group 3; a box (open box) indicates Group 4*

**Fig. 7:**
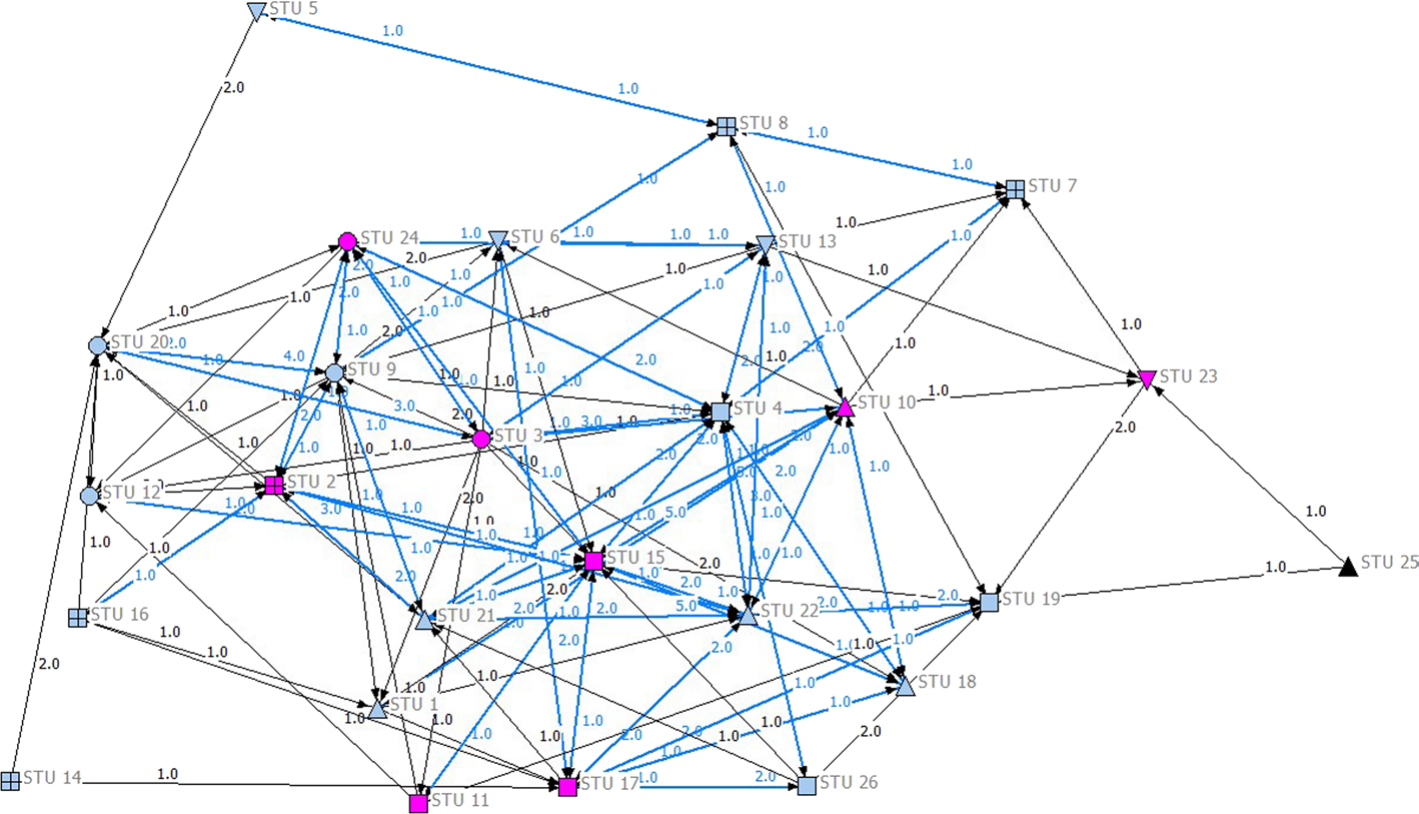
Social network interaction pattern for the GAFCC group (including original posts and peer comments). *Notes: The pink nodes indicate students with final scores from 85 to 100 (A grade); the blue nodes indicate students with final scores from 75 to 84 (B grade); the black nodes indicate students with final scores from 60 to 74 (C grade). Blue ties refer to reciprocal ties, and black ties are non-reciprocal ties. There are five study groups in this course, all formed by students exercising free choice. A circle (open circle) indicates Group 1; a square (open square) indicates Group 2; an upwards pointing triangle (open triangle) indicates Group 3; a box (open box) indicates Group 4; and a downward-pointing triangle (open downward triangle) indicates Group 5*

We conducted a social network analysis of the posts in the four online discussion forums for the two groups. The analysis results showed that the GAFCC-F and GAFCC groups’ network sizes were 23 and 26, respectively. Each of the students in the two groups contributed at least one post in the online discussion forums. The density of the GAFCC-F group was 0.289 (see Table 4), which was slightly higher than that of the GAFCC group (0.223), indicating that the GAFCC-F group had more interactions and that more participants interacted with each other in their online discussion forums. As Figs. 6 and 7 show, there were more reciprocal interactions (represented by blue ties) between two students in the GAFCC-F group than in the other group.

**Table 4:** Network size, density, and degree centralization and out-degree and in-degree centrality of the GAFCC-F and GAFCC model groups

Indicator	GAFCC-F model	GAFCC model
Network size	23	26
Density	0.289	0.223
Degree centralization	0.269	0.309
Out-degree centrality	0.601	0.509
In-degree centrality	0.4112	0.342

As shown in these figures, the GAFCC group had a higher degree of centralization (0.309) than the GAFCC-F group (0.269), indicating that the interactions in the GAFCC group were centered on fewer students, while the interactions in the GAFCC-F group involved more students. We also examined the social network interaction pattern graphs from the perspective of the students’ learning performance and group membership (i.e., intragroup and intergroup communication). Learning performance is color-coded in Figs. 6 and 7. The pink nodes represent students earning A grades (85–100 score); the blue nodes represent students with B grades (75–84 score); and the black nodes represent students with C grades (60–74 score). No students scored below 60. The node shapes represent the study groups. The students had free choice of study groups, and they selected their group mates themselves. As seen in Figs. 6 and 7, there were more interactions between students of different learning performance levels in the GAFCC-F group than in the GAFCC group. Furthermore, there were more intergroup interactions in the GAFCC-F group than in the GAFCC group. There were fewer isolated students in the interaction network in the GAFCC-F group. The GAFCC-F group had a higher value in both out-degree and in-degree centrality, indicating that students in the GAFCC-F group created more posts and gave more replies to peers’ posts (see Table 4). In summary, students in the GAFCC-F group interacted with each other more, and they contributed more equally to the interactions in the community, regardless of differences in learning performance or group membership, than the GAFCC group.

### Students’ perceptions of the GAFCC-F model

#### Students’ reflections on the fantasy element

We interviewed 15 students in Study 2 to understand their perceptions of the GAFCC-F model, particularly of the fantasy element. Overall, 87% of the participants reported their preference for narrative-based fantasy in a gamified fully online class. Three primary themes emerged from coding the transcripts: (a) *feeling in control of the process of learning*; (b) *participating more in learning activities and tasks*, and (c) *feeling the presence of peers and competition.*

Twelve students reported feeling an element of choose-my-own-adventure; as one interviewee said, “This experience is quite interesting, which provides routes for us to select the pathway. I can choose two ways or just one to complete the tasks” (Student 2). Another stated, “By viewing the story image, I can know where I am in the whole adventure, and the new unlocked plot is a recognition of my effort in completing the tasks” (Student 6). Eleven students stated that they had participated more in learning activities and tasks than in traditional class. Most of them (8 out of 11) stated that they were engaged in the unlocking function, for example, “We could unlock different tasks on each route at different times, but actually it encouraged us to go through both of the routes to complete the tasks and earn more points to beat others” (Student 1). “The engaging storyline kept me exploring the next plot by completing more and more difficult tasks.” (Student 3). Six students stated that they kept exploring the next plot by completing the tasks. “The gamified narrative engaged me and made me really want to know what will happen next and the end of this narrative” (Student 8). “‘Save Princess Joanne’ is very interesting; it’s really like an adventure.… [T]he location of four characters in each level is different; I did more tasks to see the ending of the narrative” (Student 5).

Four students reported that they appreciated the use of a new plot image to acknowledge their participation; for example, “Images of levels showing my character in different locations were helpful, acknowledging our efforts and our achievement in learning” (Student 11); “It can really have an impact on boosting my confidence, as a recognition of my participation performance. It can bring positive psychology to my studies” (Student 10). Nine students also stated that although it was a fully online teaching mode, they nonetheless felt the presence of peers and competition in this gamified class. “Our group worked so hard to get the real formal reputation or honor to defeat other groups in terms of total earned points” (Student 14). “We [needed] to compete with other groups and gain a higher score. Our group members were so excited to do our best throughout the semester” (Student 15).

#### Students’ suggestions for the narrative design of future gamified classes

We also gathered students’ suggestions for improving the fantasy design. There were two main suggestions. The first suggestion was to consider an interactive narrative wherein a variety of subsequent scenarios are triggered by the learners’ actions, meaning that a scenario is contingent on the performance of prior tasks or based on a learner’s choice (i.e., allowing learners to pick the plot that most interests them). This would permit individual learners to access different sets of scenarios and would arouse their curiosity, leading them to explore more plots by completing more tasks. The second suggestion was to relate scenarios in the narrative to course content. Although the “Save Princess Joanne” narrative offered a very intriguing and joyful learning experience, combining the scenarios with the course content could have appealed more to adult learners. For example, the course of study included in this course could offer a full narrative about how an instructional designer intern deals with different clients’ requests. This highlights the importance of taking educational contexts (e.g., K–12 or adult education) into the design of the narrative.

### Teachers’ perceptions on the GAFCC-F model

#### Teachers’ reflection on the fantasy element

Three themes were generated based on the teachers’ reflections on the use of fantasy in the online gamified learning design: (a) *connecting learning tasks with different levels of difficulty*, (b) *unlocking learning tasks in sequence using a linear structured narrative*, and (c) *providing a joyful virtual learning experience*. These are described in more detail below.

Fantasy helps to connect different learning tasks to different levels of difficulty. The teachers stated that the narrative was designed to make the unlock function entertaining to learners. As one noted, “As they complete a simple task in a scenario, a much more difficult task is unlocked in a continuous scenario. This helps learners immerse themselves in the virtual world and monitor their progress in task completion by checking how many scenarios have been shown so far” (Teacher 1). Another commented:

The unlock scheme is rooted in the linear structure of the narrative.

[The] “Save Princess Joanne” narrative is a linear adventure game that shares many features with [the] Mario Adventure game. Players are encouraged to move forward … with … increasingly accumulating points. Thus, the past unlocked scenarios would not affect their task performance in the forwarding new scenarios. Also, no points deduction was involved for [unsatisfactory] performance. By using [a] linear narrative, we aim to promote students’ motivation in making more attempts on individual tasks with less fear of failure (Teacher 1).

The teachers also stated that the linear narrative suits the linear layout of the Moodle platform. Embedding each scenario in Moodle allowed students to flip forward and backward through the unlocked scenarios like a book.

The teachers reflected that fantasy helped amplify the impact of the unlocking element in gamification by providing a linear forward narrative and an imaginary world. The newly unlocked plot gave meaning to task completion, for instance, submitting a group project meant slaying a small blob enemy and completing a difficult quiz meant finding out where the princess was being detained. This response corresponds with Werbach’s (2012) comment that adding narratives in gamification is helpful in arranging game elements in a coherent way to present a meaningful, ongoing context for the players.

Furthermore, fantasy helped to provide a joyful virtual learning experience. “Because of the COVID-19 pandemic, the course was delivered in [a] fully online mode. Many students lost the chance of physically visiting City K; thus, we inserted City K local culture elements into the narrative design” (Teacher 2). The teachers explained why they had chosen the sightseeing attractions in City K as the background images in the narrative. “Students really appreciated this design to make up their virtual experience of studying and living in City K” (Teacher 2).

#### Teachers’ suggestions for the narrative design of future gamified classes

The teachers also gave suggestions for improving the online gamified class design. They reported that in addition to satisfying the concept of “fantasy,” the narrative in gamified learning must represent real-world scenarios. The “Save Princess Joanne” narrative resembles the mechanism of the game Mario Adventure, in which the player is completely detached from the game character in the pixel world. Although there was a final goal in the narrative, saving the princess, this goal was not related to the course content. Such scenarios may not interest every adult learner. It was suggested by the students that real-world instructional designer scenarios be used to create a risk-free but realistic world simulation to expose learners to a virtual instructional design workplace. This would allow learners to practice skills and knowledge in a virtual setting, to make mistakes and explore the best path to succeed without their performance’s impacting a real business. By aligning the setting of game scenarios with the course content, teachers can offer their students a joyful learning experience that reflects the skills and knowledge required in the course. Students could even develop instructional design product portfolios after completing the tasks in the gamification. This would add value to their real-world life, for example by providing materials relevant to job applications.

## Discussion

### Students' and teachers' reflections on the original GAFCC model and suggested refinement to the model

In this section, we present students’ and teachers’ reflections on the original GAFCC gamification design model in Experiment 1 to explore the need of refining this model (if any). Although a large majority of students preferred the use of gamification to non-gamified courses, more than half of them gradually lost interest when the semester progressed. As the interview results showed, most students reported only a short duration of engagement in the GAFCC gamified class. Students perceived a high level of engagement during the first half of the semester, especially for students who had their first taste of gamification. But when they became familiar with the gamification scheme later, they gradually lost interest and participated less in the online activities (i.e., online discussion, quizzes), a phenomenon that we have labelled here as the novelty effect (Clark, 1983). The teachers similarly observed that students’ participation in the in-class activities decreased over the 10 sessions. The problem of the novelty effect has been documented in various bodies of literature, from the introduction of new information technology systems to gamification systems (Hamari et al., 2014). In the context of gamification, user engagement gradually disappears when the game elements no longer satisfy them, a phenomenon also known as the hedonic treadmill (Brickman & Campbell, 1971). Therefore, there is a crucial need to enhance students’ motivation in completing course activities by using other means such as fantasy. Fantasy can increase learners’ motivation by making the learning more fun (Malone, 1981).

### Incorporating fantasy into the GAFCC model and its effects on students’ learning performance and online social interaction

#### Impact on students’ learning performance

Previous research has revealed that students with a higher level of motivation tend to have satisfactory academic achievement (e.g., Badri et al., 2014). In the GAFCC-F gamification design model, students’ learning performance was significantly higher than that of the students in the GAFCC model in terms of mid-term and post-test scores. Islas Sedano et al. (2013) found that fantasy is the central factor in educational games’ ability to trigger learners’ affective and cognitive engagement. Fantasy may better immerse learners in the learning environment and may help them improve their learning (Islas Sedano et al., 2013). In other words, narrative-based fantasy helps immerse learners in the context and thus may increase their engagement. As student engagement is an important indicator of learning performance (Qureshi et al., 2021), the GAFCC-F model, which provides a more engaging fully online learning context than a model without fantasy, is likely to correlate with a higher level of learning performance, especially in terms of students’ application of learnt skills and knowledge.

#### Impact on students’ online social interaction

Students in the GAFCC-F group showed higher levels of thinking in their online posts than students in the GAFCC group. They displayed more elaboration, reflection, and evaluation in their online comments than the other group. Moreover, their posts displayed higher knowledge quality and a greater significance of findings than their counterparts. We found that the students in the GAFCC-F group engaged in more online discussions with peers across study groups and peers of different levels of learning performance than the students in the GAFCC group.

Narrative-based fantasy can facilitate players’ adoption of the perspectives and emotions of the actors in the narrative (Lee et al., 2016). Players tend to internalize the messages delivered in a game and change their own behaviors accordingly (Slater & Rouner, 2002). Participants who identify and perceive similar feelings to those of narrative characters are especially prone to these behavioral changes in the long term (Hui et al., 2021). A meta-analysis also revealed that interventions based on narrative-based fantasy games had a small effect on the players’ behavioral change (Cohen’s *d* = 0.32). Specifically, we observed continuous engagement in the form of posting and interacting with peers in online discussion forums. By providing vivid images and using personal stories to convey information, narrative-based fantasy enables people to be transported into the storyline (Kim et al., 2012) and increases their attentional focus (Green, 2006). In other words, it enhances their affective engagement in a certain environment. Therefore, the “Save Princess Joanne” narrative-based fantasy immersed the students in a virtual context and motivated them to complete more learning tasks to accomplish the final goal in the story, to bring the princess back.

### Students’ and teachers’ perceptions of the GAFCC-F model

In this study, the fantasy environment was constructed by presenting a game-like story using a first-person perspective. Because of the two routes in the “Save Princess Joanne” story, supported by an unlocking mechanism, some students reported that they could keep experiencing new learning settings, bringing about a sense of playing a game. Furthermore, the students earned badges for the 12 levels in the fantasy world. These badges also depicted different locations, based on where the characters (represented by the students) obtained various clues for finding the princess. This helped enhance students’ sense of identification in the game. A player’s identification in a game comprises their ability to experience the characters’ feelings, adopt the character’s viewpoint, and internalize the character’s missions (Qureshi et al., 2021). Such identification is crucial for providing learners with a good learning experience and increasing their engagement in the game environment (Hui et al., 2021). In addition, we delivered the whole story in the first-person perspective, which can help students maintain greater enjoyment and engagement with the activity than a third-person perspective (Bickmore et al., 2010). To summarize, the students were content with the gamified learning experience supported by the GAFCC-F model.

From the teachers’ perspective, the “Save Princess Joanne” story itself can be regarded as exogenous or extrinsic fantasy (Malone, 1981). Exogenous fantasy does not require changes in the storyline when applied to another context (Kenny & Gunter, 2007). Thus, according to the course teachers in this study, this fantasy could be easily incorporated into any other courses so long as there are clear learning objectives to achieve. The learning objectives can be set as the final mission (i.e., to slay the bad dragon in the “Save Princess Joanne” story) in the story. The course teachers felt that the exogenous fantasy environment can be easily replicated, and its impact can be examined in many other courses. This is important because perceived ease of use has a direct impact on people’s intentions to use a tool or system (He et al., 2018). In other words, if a user feels that using the exogeneous fantasy is free of effort, the user will be more willing to use it in his or her course.

### Limitations and future work

This study has two limitations that must be acknowledged. First, we examined the GAFCC-F model in the context of an e-learning strategy and management course focused more on the application of learnt skills and knowledge than on concepts. Findings may differ in theoretical subjects such as math and physics, which rely heavily on verifiable concepts and principles used to explain phenomena (Ruengtam, 2012). Second, in light of the unbalanced ethnic composition of the participants in the two studies (i.e., 89.8% of the participants come from East Asian regions), this study mainly represents the context of East Asian students in higher education. Research has suggested that the effect of perseverance of effort on learning achievement is more positively associated with East Asian cultures than with Western cultures (Xu et al., 2021); that is, East Asian learners may be more likely to display satisfactory learning performance in a new learning context (in our case, a fully online class).

Therefore, options for future research include testing the GAFCC-F gamification design model in a other subject disciplines, examining the effects of this model in an online class of students from other cultural backgrounds, and using collaborative online platforms (e.g., Miro, an online whiteboard tool) to track students’ performance in the in-class activities.

## Conclusion

Although the COVID-19 pandemic has forced many higher education institutions to conduct their lessons via the Internet, fully online learning is still regarded as being less engaging than in-person teaching. Against this backdrop, this study tested and refined a theoretical based GAFCC gamification model and compared the effects of the original GAFCC model with those of a GAFCC-F model on students’ learning performance and online social interaction, as well as the students’ and teachers’ perceptions of the educational experience.

The following two implications for fully online learning may be inferred from this study. First, we found overall empirical and theoretical support for the use of the GAFCC-F model in the context of fully online learning. Specifically, we found that the GAFCC-F model was more effective in improving the students’ learning performance, enhancing online social interaction with peers across different study groups, and providing a joyful learning experience than the original GAFCC model. The implication here is that the inclusion of fantasy into gamification can promote student learning and quality of online interaction. The GAFCC-F model therefore offers instructors a possible solution to address the problem of student disengagement in fully online learning courses. In particular, the use of exogeneous fantasy provides a form of motivational embellishment that can be easily added without requiring changes in the basic instructional content of the course activities. This permits easy incorporation of a fantasy context by a course teacher into other courses.

Second, despite the overall positive support for the use of the GAFCC-F model, both students and course teachers recommend that the fantasy narrative in gamified learning should represent real-world scenarios that relate more closely to the course contents. The implication here is that although the “Save Princess Joanne” fantasy narrative used in this study offered an intriguing and joyful learning experience, associating the fantasy story to the course content could appeal more to adult learners. This highlights the importance of taking educational contexts (e.g., K–12 or adult education) into the design of future fantasy narrative to engage students in fully online learning environments.
